# The effect of age-related macular degeneration on cognitive test performance

**DOI:** 10.1038/s41598-022-07924-8

**Published:** 2022-03-08

**Authors:** Anne Macnamara, Victor R. Schinazi, Celia Chen, Scott Coussens, Tobias Loetscher

**Affiliations:** 1 Cognitive Ageing and Impairment Neurosciences Laboratory, Justice and Society, University of South Australia, Adelaide, SA Australia; 2 Department of Psychology, Faculty of Society and Design, Bond University, Gold Coast, QLD Australia; 3 Future Health Technologies, Singapore-ETH Centre, Campus for Research Excellence and Technological Enterprise (CREATE), Singapore, Singapore; 4 Department of Ophthalmology, Flinders Medical Centre, Flinders University, Adelaide, SA Australia

**Keywords:** Neuroscience, Psychology, Health care

## Abstract

The reliable assessment of cognitive functioning is critical to the study of brain-behaviour relationships. Yet conditions that are synchronous which ageing, including visual decline, are easily overlooked when interpreting cognitive test scores. The purpose of this study was to demonstrate the negative consequences of visual impairments on cognitive tests performance. Moderate to severe levels of age-related macular degeneration were simulated, with a set of goggles, in a sample of twenty-four normally sighted participants while they completed two cognitive tasks: a vision-dependent reaction time task and a vision-independent verbal fluency test. Performance on the reaction time task significantly decreased (*p* < 0.001) in the simulated age-related macular degeneration condition, by as much as 25 percentile ranks. In contrast, performance on the verbal fluency test were not statistically different between the simulated and normal vision conditions (*p* = 0.78). The findings highlight the importance of considering visual functioning when assessing cognitive function. When vision is not accounted for, low test scores may inaccurately indicate poor cognition. Such false attributions may have significant ramification for diagnosis and research on cognitive functioning.

## Introduction

Cognitive tests scores inform research and diagnoses in aging and neurodegenerative disorders. However, these scores can be impacted by a range of factors not directly measured by tests, ranging from situational, personal, language to cultural factors^1^. While some of these factors may be easier to identify, others are more elusive. For example, a language barrier can become quickly apparent if participants struggle to understand instructions or perform adequately on a written task. On the other hand, impairments of a visual nature can be harder to recognise as there may be no clear indication of impaired visual function^2^. Indeed, vision impairments are often overlooked in research and clinical settings; it has previously been estimated that reduced vision may be undetected in up to 50% of older adults^3^.

In 2020, moderate to severe vision impairment affected approximately 200 million people over the age of 50^4^. Given that the prevalence of visual impairment is only estimated to increase due to the aging population, researchers and clinicians focusing on ageing and neurodegenerative disorders need to pay close attention to the possibility that visual impairments may affect the scores of cognitive tests. One leading cause of visual impairment is age-related macular degeneration (AMD), which may result in an irreversible loss of central vision^5^; and can negatively impact tasks involving visual functioning including reading, driving and recognising faces^6^. Critically, AMD is known to be underdiagnosed in the elderly, with an estimated 25% of eyes medically judged to be ‘normal’, actually having features of AMD and suboptimal vision^7^.

To highlight the importance of central vision for cognitive assessments, we simulated visual impairment with AMD simulation goggles, while participants completed a series of cognitive tasks. Since older adults with visual impairments are significantly more vulnerable to physical and mental comorbidities (i.e., Parkinson’s disease, dementia, hearing loss), a vision loss simulation with healthy, normally-sighted participants can more easily isolate vision-related effects on behaviour^8,9^. While simulations may never wholly replicate a visual impairment (e.g., due to patient variability in symptom presentation; underdeveloped compensatory strategies; and lack of progressive visual decline)^9^, thus far simulating vision loss has been a simple, yet valid approach to investigate the effects of visual impairments on cognition^10,11^. Furthermore, AMD simulations have replicated patterns of behaviour and difficulties experienced by AMD patients^9^.

## Methods

### Participants

An a priori power calculation was conducted to estimate the required number of participants for a larger study investigating the effects of simulated AMD on anxiety and stress levels in everyday activities (not reported here). Using G*Power^12^, it was estimated that a minimum of 13 participants were required to provide sufficient power (0.90), at a significance level of α = 0.05, to detect a large effect. A large effect could increase the practical significance of the findings. The estimate was based upon an AMD simulation study which was similar in nature to the larger study conducted^13^. All eligible participants that signed up during the advertisement period (between July and September 2020) were tested. Data were only analysed after data collection was completed.

Twenty-four normal-to-corrected sighted (best corrected visual acuity of greater > 6/18) participants (19 women) aged 18–60 (Mean = 27.1, SD = 9.7) completed the experiment. They were English speakers, and had no history of visual impairment, anxiety disorders, psychiatric disorders, or cognitive impairment. Participants were recruited at the University of South Australia (UniSA) and via the UniSA online research participation system, and informed consent was obtained from all. The study was approved by the UniSA Human Research Ethics Committee (Ethics Protocol 202889); and it was conducted in accordance with the Declaration of Helsinki, and the Australian National Statement on Ethical Conduct in Human Research guidelines.

### Apparatus and materials

**Figure 1:**
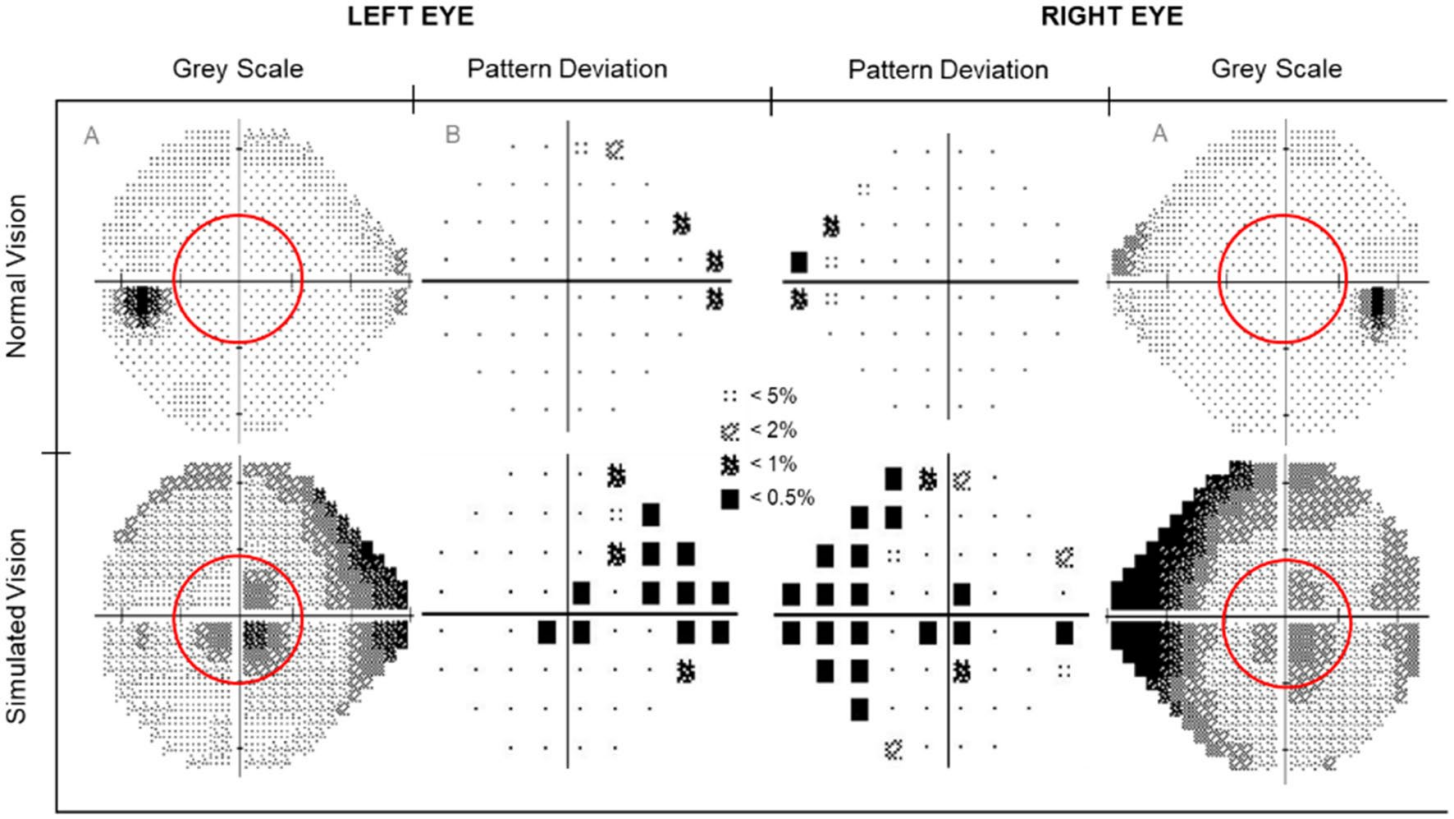
Results of the visual field test. Top row: normal visual field in the left and right eye. Bottom row: simulated macular degeneration visual field with a central 10° scotoma (red circles).

The visual effect of AMD was induced with enhanced Fork in the Road Macular Degeneration simulator goggles^14^. The severity was manipulated to reflect moderate to severe AMD—visual acuity 6/18 to 3/60 respectively^15^. The goggles were enhanced by the addition of two layers of 20 mm diameter circular Bangerter occlusion foils of 0.1 LogUnit (resulting in 20/200 or 6/60 vision), positioned in the central inner region of each lens. These enhancements were calibrated by a neuro-ophthalmologist to ensure the simulator resulted in a reduction in best corrected visual acuity to 6/60 and created a 10° central scotoma monocularly in each eye. The visual acuity was verified using Snellen linear acuity at 6 m and the scotoma was confirmed with a Zeiss Humphry 24-2 automated visual field analyser (see Fig. 1; Carl Zeiss Meditec, Inc. Jena, Germany). To ensure the results were not confounded by the goggle frames (e.g., restricted peripheral vision), identical goggles with clear lenses were worn in the normal vision condition. If required, participants wore prescription glasses under the goggles.

Cognition was assessed via a vision-dependent Reaction Time Task (RTI)^16^ and a vision-independent Verbal Fluency Test (VFT)^17^. The tests were chosen as they are suitable for assessing cognition in aging, clinical populations (e.g., Alzheimer’s)^18–21^. The RTI [choice], from the Cambridge Neuropsychological Test Automated Battery (CANTAB)^16^, assesses mental and motor response speeds. Participants pressed a button on the screen, after which a yellow dot appeared in one of five circle locations. Participants were instructed to move their finger from the button to the yellow dot, as quickly and accurately as possible. Mental responses reflected the times taken for participants to identify the yellow dot location and release the button. Motor responses were the times taken for participants to move from the button to the yellow dot.

The VFT appraises semantic and phonemic fluency^17^. Participants had sixty seconds to generate as many different words (excluding names, places, and repeated words with different endings), starting with the letter F or S. The VFT was conducted as a control task, because unlike the RTI, it does not require vision for completion.

### Procedure

**Figure 2:**
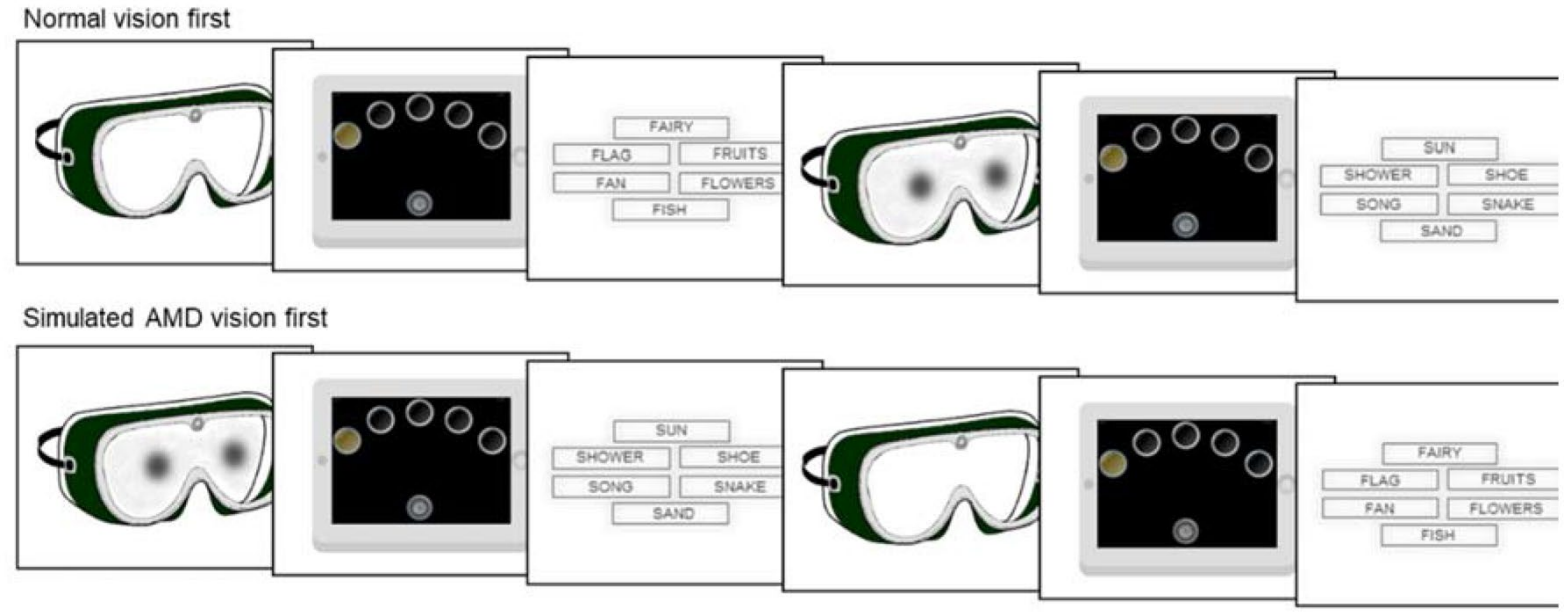
Participants completed the reaction time task and verbal fluency test under their assigned vision condition (normal or simulated AMD), then repeated the tasks under the opposite vision condition.

Participants completed each cognitive task twice, under normal and simulated AMD vision (see Fig. 2). The VFT starting letter (F or S), order of cognitive task (RTI or VFT) and order of vision condition (normal or simulated AMD) were counterbalanced across participants.

### Statistical analysis

We conducted separate 2 (visual condition: normal or simulated AMD) × 2 (order: normal or simulated AMD vision first) repeated measures ANOVA using jamovi^22^ for the mental and motor responses in the RTI, and the VFT.

The TOSTER module^23,24^ in jamovi with equivalence bounds of ± 0.5 Cohen’s d_z_ and an alpha of 0.05 was used to test for equivalence between the two vision conditions in the case of non-significant results in the above analyses.

To further quantify the impact of AMD on cognitive performance, the cNORMJ module^25^ in jamovi estimated T-scores based on the results of the normal vision condition. Using an inverted ranking order, a quartic polynomial regression modelled the relationship between raw and norm scores. A norm table for normal vision was compiled based on the model, and changes in percentile ranks for the simulated AMD condition was calculated.

## Results

**Figure 3:**
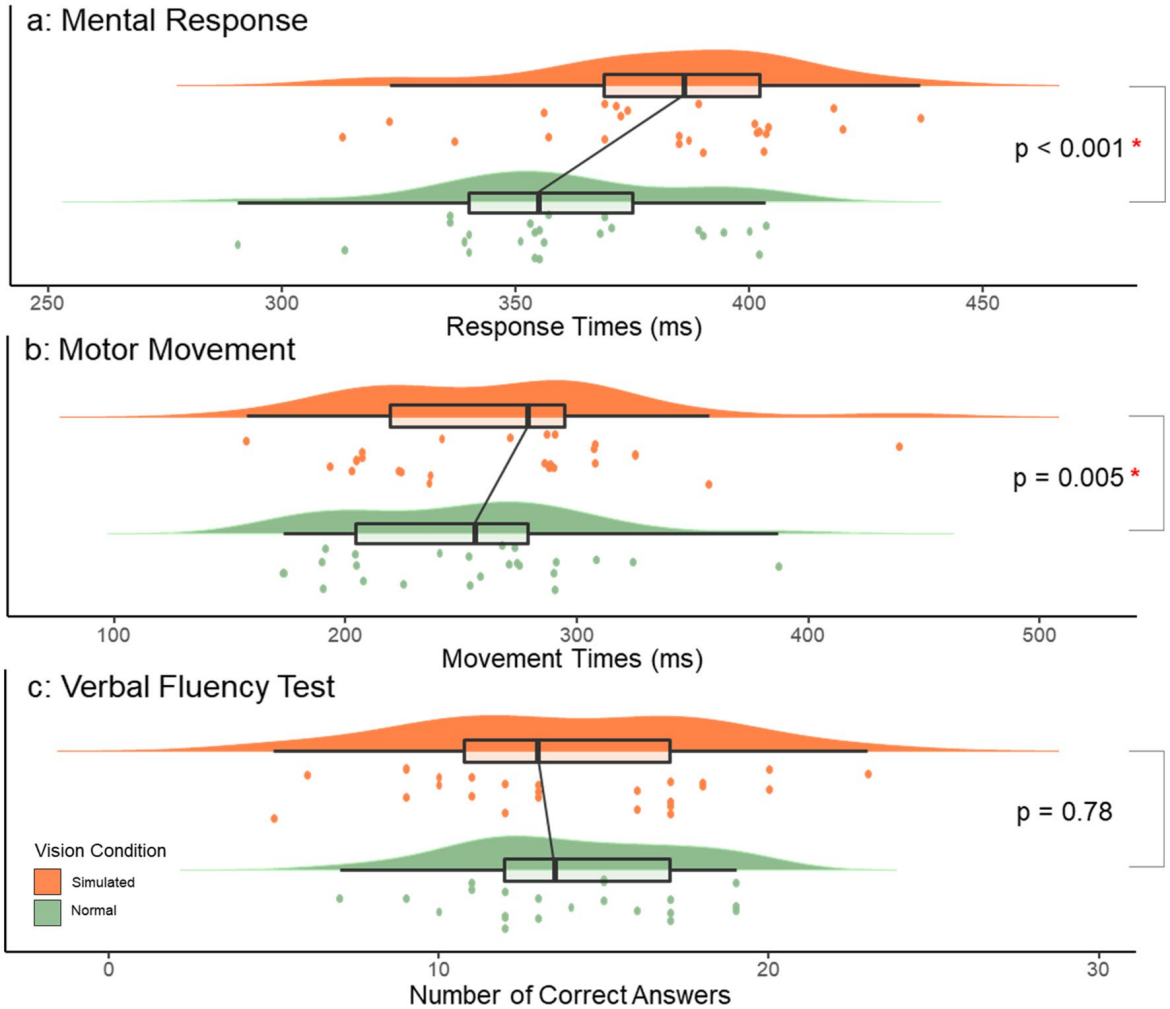
Normal and simulated AMD vision results. (**a**) RTI mental responses. Main effect of vision (*p* < 0.001). (**b**) RTI motor movements. Main effect of vision (*p* = 0.005). Interaction between vision and order (*p* = 0.01). (**c**) VFT correct answers. Main effect of vision (*p* = 0.78). Significance level: *p* < 0.05.

For the RTI, mental response times significantly increased in the simulated AMD condition (381.98, SD = 29.90 ms) compared to the normal vision condition (359.02, SD = 28.04 ms; see Fig. 3), (*F*(1,22) = 31.66, *p* < 0.001, *n*^2^_p_ = 0.59). There was no main effect of order (*F*(1,22) = 1.01, *p* = 0.33, *n*^2^_p_ = 0.04) or an interaction between vision and order (*F*(1,22) = 0.315, *p* = 0.58, *n*^2^_p_ = 0.01).

For the RTI motor responses, there was also a main effect of vision (*F*(1,22) = 9.65, *p* = 0.005, *n*^2^_p_ = 0.30), with slower movement times in the AMD (265.67, SD = 61.79 ms) compared to the normal vision (250.56, SD = 52.87 ms) condition. There was no main effect of order (*F*(1,22) = 0.88, *p* = 0.359, *n*^2^_p_ = 0.04), but a significant interaction between vision and order (F(1,22) = 8.07, *p* = 0.01, *n*^2^_p_ = 0.27). Simple main effects revealed no difference between vision conditions if participants started with normal vision (*p* = 0.859). However, if they completed the RTI first with the AMD goggles, their motor responses were significantly faster when they subsequently did the task with normal vision (*p* < 0.001).

In the VFT, there were no main effects of vision (F(1,22) = 0.079, *p* = 0.78, *n*^2^_p_ = 0.004), order (F(1,22) = 1.21, *p* = 0.28, *n*^2^_p_ = 0.05), nor an interaction (F(1,22) = 1.40, *p* = 0.25, *n*^2^_p_ = 0.06). Equivalence testing confirmed there were no meaningful differences in the VFT as a function of visual condition (*t*(23) = 2.17, *p* = 0.017).

## Discussion

Our findings provide a compelling demonstration of how visual impairments may significantly impact performance on cognitive tasks that rely on vision. The RTI was compromised due to the AMD simulation, yet the VFT remained unaffected. To put the findings into the context of standardized scores, the mean mental response time for the simulated AMD condition in the RTI was approximately 25 percentile ranks lower than in the normal vision condition. Being scored in the 25th percentile instead of the 50th percentile, as in our study, is a significant reminder to researchers that the added interference due to vision loss deserves attention and should not be easily discounted^26^.

Even though cognitive tests are just one aspect of the diagnostic process, the inaccurate scoring of cognitive performance could still contribute towards the misdiagnosis of cognitive related problems, including mild cognitive impairment (MCI) or dementia. In this event, subsequent issues can arise. For example, a mistaken diagnosis of dementia may precipitate unnecessary changes to a person’s living, working, financial or social circumstances^27^. Furthermore, the diagnosis of MCI can trigger psychological problems (e.g., depression and anxiety) due to the stigma of cognitive impairment^28^. For people with AMD, who are already experiencing physical and psychological issues due to vision loss^6,29^, the multitude of repercussions that inaccurate cognitive assessments causes are an unneeded additional burden.

It only takes the incorporation of simple precautionary measures in order to make allowances for the potential impact of AMD. For example, screening participants with mobile vision charts (e.g., Snellen)^30^ prior to participation, or administering vision-friendly variations of standard cognitive assessments (e.g., blind MOCA)^31^. While our findings specifically relate to AMD, the differences between normal and simulated conditions corroborate previous studies using paper-and-pencil tests under low visual acuity or cataract simulations^10,11,32^. The findings also align with studies assessing cognition in older clinical populations, indicating that this problem is systematic across a range of visual impairments^33,34^.

It is currently unclear whether these simulations lead to an over- or underestimation of the true impact of visual impairments on test performance, but there are reports that the severity of AMD health effects are underestimated with lenses that simulate AMD^35^. While the true impact of AMD on cognitive test scores remains to be established, it is clear that not controlling for vision can adversely affect the results and can have broader implications for the health of visually impaired people.

## Data Availability

The datasets generated during and/or analysed during the current study are available from the corresponding author on reasonable request.
